# Involvement of a Case Manager in Palliative Care Reduces Hospitalisations at the End of Life in Cancer Patients; A Mortality Follow-Back Study in Primary Care

**DOI:** 10.1371/journal.pone.0133197

**Published:** 2015-07-24

**Authors:** Annicka G. M. van der Plas, Kris C. Vissers, Anneke L. Francke, Gé A. Donker, Wim J. J. Jansen, Luc Deliens, Bregje D. Onwuteaka-Philipsen

**Affiliations:** 1 Department of Public and Occupational Health, VU University Medical Center, Amsterdam, the Netherlands; 2 Center of Expertise in Palliative Care, VU University Medical Center, Amsterdam, the Netherlands; 3 EMGO Institute for Health and Care Research, VU University Medical Center, Amsterdam, the Netherlands; 4 Department of Anaesthesiology, Pain, and Palliative Medicine, Radboud University Nijmegen Medical Centre, Nijmegen, the Netherlands; 5 Nursing Care, NIVEL Netherlands Institute for Health Services Research, Utrecht, the Netherlands; 6 NIVEL Primary Care Database, Sentinel Practices, Utrecht, the Netherlands; 7 Department of Anaesthesiology, VU University Medical Center, Amsterdam, the Netherlands; 8 End-of-Life Care Research Group, Vrije Universiteit Brussel and Ghent University, Brussel and Ghent, Belgium; Nathan Kline Institute and New York University School of Medicine, UNITED STATES

## Abstract

**Background:**

Case managers have been introduced in primary palliative care in the Netherlands; these are nurses with expertise in palliative care who offer support to patients and informal carers in addition to the care provided by the general practitioner (GP) and home-care nurse.

**Objectives:**

To compare cancer patients with and without additional support from a case manager on: 1) the patients’ general characteristics, 2) characteristics of care and support given by the GP, 3) palliative care outcomes.

**Methods:**

This article is based on questionnaire data provided by GPs participating in two different studies: the Sentimelc study (280 cancer patients) and the Capalca study (167 cancer patients). The Sentimelc study is a mortality follow-back study amongst a representative sample of GPs that monitors the care provided via GPs to a general population of end-of-life patients. Data from 2011 and 2012 were analysed. The Capalca study is a prospective study investigating the implementation and outcome of the support provided by case managers in primary palliative care. Data were gathered between March 2011 and December 2013.

**Results:**

The GP is more likely to know the preferred place of death (OR 7.06; CI 3.47-14.36), the place of death is more likely to be at the home (OR 2.16; CI 1.33-3.51) and less likely to be the hospital (OR 0.26; CI 0.13-0.52), and there are fewer hospitalisations in the last 30 days of life (none: OR 1.99; CI 1.12-3.56 and one: OR 0.54; CI 0.30-0.96), when cancer patients receive additional support from a case manager compared with patients receiving the standard GP care.

**Conclusions:**

Involvement of a case manager has added value in addition to palliative care provided by the GP, even though the role of the case manager is ‘only’ advisory and he or she does not provide hands-on care or prescribe medication.

## Introduction

The aim of palliative care is to improve the quality of life of patients and their families facing the problems associated with life-threatening illness, as stated in the World Health Organisation (WHO) definition [1]. Most people prefer to die at home [2] and home is also considered to be the preferred place of care at the end of life. A high percentage of patients with home deaths and a low number of hospitalisations are considered outcomes of high quality palliative care [3;4]. Therefore, the availability of community-based palliative care is important in enabling patients’ palliative care wishes and needs to be met.

In the Netherlands, the general practitioner (GP) and home-care nurse are main care providers for patients with palliative care needs living in the community. The number of non-sudden deaths per GP per year is estimated to be 12 to 13 on average [5]. Home-care nurses and home support workers who are confronted with end-of-life care see on average 10 palliative care patients a year [6]. Patients have a broad range of symptoms and it is hard to keep up to date with the new, advanced and complex treatment options now available in palliative care [7–9]. Nurse case managers with specific expertise regarding palliative care have been introduced in some regions to help patients and their informal carers obtain the palliative care that matches their preferences. Most patients are referred to the case manager early in the palliative care trajectory and they are mostly referred by hospital staff (62% of referrals) [10]. The majority (69%) of patients referred to a case manager received a combination of curative or life-prolonging treatment and palliative care [10]. To ensure continuity of care, a case manager collaborates with the patient, their informal carers and the professionals involved in care for the patient, such as the GP or the medical specialist [11]. The case manager provides advice to patients and their informal carers and refers them to other care providers when necessary. Additionally, the case manager may offer advice and information about good palliative care to other healthcare providers involved with the patient, mostly the GP and the home-care nurse.

A literature review has shown that specialised palliative care at home increases the chance of dying at home and reduces symptom burden, in particular for patients with cancer [12]. However, a generalist palliative care model can also result in good quality palliative care as indicated by a low percentage of patients with hospitalisations in the last month of life [13]. For sustainable palliative care in an aging society, it is argued that basic palliative care should be provided by generalist healthcare professionals and that specialist palliative care should be reserved for more complex situations [14]. This is the care model that is used in the Netherlands.

It is unclear whether there is additional value in having a case manager for patients with palliative care needs. Therefore, in this paper we compare patients primarily receiving palliative care from their GP alone with patients who were also referred to a case manager for additional support. The following data were compared: 1) the patients’ general characteristics, 2) characteristics of care and support given by the GP (number of patients with contact with their GP, number of contacts between the patient and the GP, involvement of a home-care nurse and palliative care consultant other than the case manager), 3) palliative care outcomes (preferred place of death is known by the GP, place of death, number of transfers, number of hospitalisations in the last 30 days).

## Methods

### Setting

The population of the Netherlands is 16.9 million [15]. Each year, about 77,000 people die of non-acute illnesses, 31% of them dying at home [16]. Almost all Dutch residents are registered with a GP, who functions as a gatekeeper for more specialised forms of care. Palliative care is part of the educational programme for GPs and home-care nurses, and there are also a wide range of short courses available on palliative care. Fewer than 1% of GPs and home-care nurses have had advanced education to specialise in palliative care [5]. Specialised palliative care knowledge is available to GPs and home-care nurses through consultation teams operating all over the Netherlands, mainly offering advice by telephone. Nurse case managers with specific expertise in palliative care who visit patients at home have also been introduced in some regions (for a map of the Netherlands showing which regions, see [17]).

Case management is provided by a nurse with expertise in palliative care who functions as a case manager [17]; he or she visits the patient and their informal carers at home to offer support and advice on care and treatment options. The case manager monitors whether care is being delivered according to the patient’s and informal carers' wishes and needs. Information and psychosocial support are provided by the case manager if patients and their informal carers wish so. The case managers do not provide hands-on nursing care themselves but can be part of a team that does. Most case managers (62%) were trained in nursing at the bachelor level with further education in oncology or another relevant field of specialist care. The organisational affiliation of the case managers varies; case managers can be employed by a home-care organisation, by a hospice or by a collaborative venture between institutions (e.g. a home-care organisation working together with a hospital). Detailed information on the content of the support provided by case managers can be found elsewhere [18]. There are case managers in the Netherlands for patients with dementia [19], but they are not included in this paper.

### Design and sample

This article is based on questionnaire data provided by GPs participating in two different studies: the Sentimelc study [20] and the Capalca study. The Sentinel-Monitoring End-of-Life Care (Sentimelc) is a mortality follow-back (retrospective) study. It provided the data for this paper on standard GP care. The Capalca study is a prospective study. It provided the data on care where case managers were involved. Both studies were conducted within the same research team, and several questions were made to match to enable the comparison of the data from the Capalca and Sentimelc studies.

#### Standard GP Care

The aim of the Sentimelc research project is to monitor the quality of care provided by GPs to a general population of end-of-life patients in the Netherlands. Data were collected via the Sentinel practices in the Nivel Primary Care Database, a pre-existing continuous monitoring system based on a representative sample of GPs reporting on several diseases and interventions [20]. For this paper end-of-life data from 2011 and 2012 were analysed.

#### Care where case managers were involved

The Capalca study was set up to investigate the implementation and outcomes of the support provided by case managers in primary palliative care. A nationwide survey was conducted to identify initiatives involving case managers [17]. The term ‘initiative’ is used to do justice to organisational differences, since not all case managers work in a team of case managers; there was one initiative with one case manager, for example, while another case manager was part of a team in which not all members offer case management. Of the 20 initiatives identified in that survey, 13 were investigated in this paper. Case management as provided to the patient was monitored prospectively by questionnaires. Case managers who support many patients could include every second patient in the Capalca study instead of every patient (i.e. half of the patients who received support from the case manager were included in the study), for time management reasons. Data were gathered from March 2011 until the end of 2013.

The following criteria were used to select data from the two studies that were suitable for a comparison: the patients’ age was at least 18, the patients had not died suddenly and unexpectedly (the study on standard GP care) and had died during the data collection period (the study on case managers), their place of residence was ‘at home’ or ‘with informal carers’, and patients did not receive support from a case manager (the study on standard GP care). Furthermore, only cancer patients were included since the main diagnosis is expected to influence the care provided and the diagnosis composition differed between the two samples.

### Ethics statement

Under Dutch law, both the Sentimelc and Capalca studies are exempt from approval from an ethics committee. Ethical approval was not required since the studies did not involve imposing any interventions or actions [21] and posthumous collection of anonymous patient data is allowed in the Netherlands [22;23]. We have not requested a waiver from the ethics committee. All data from both the Capalca and Sentimelc study, was anonymised before being handed over to the authors. The researchers in the study on case managers did not interact with the patients. The case managers informed their patients that they were collecting information on care provision. To facilitate this, the researchers provided information material about the research project that the case managers could hand to their patients.

### Questionnaires and procedure

#### Standard GP Care

Within one week of reporting a patient’s death, participating sentinel GPs were asked to fill in a registration form surveying information regarding the care the deceased received in the last three months of life. On completion, the registration forms were returned to NIVEL where they were scrutinised for missing data and errors, duplicated and then sent to the researchers for analysis. The questionnaire included structured questions on the following: the patient’s age and sex, main diagnosis, place of death, whether the GP was aware of the preferred place of death, the places of care in the three months before death and the number of days spent per place of care, the number of contacts (home visits and consultations) in the last week, in weeks two to four, and in months two and three before death, and the involvement of other care providers. In order to clearly identify which patients would have qualified for palliative care in their final days and which not, GPs were asked if the death in question had been both ‘sudden and totally unexpected’.

#### Care where case managers were involved

If a patient was referred for case management, the responsible case manager filled in a questionnaire. After the patient’s death, the case manager sent a questionnaire to the GP. The two questionnaires used the same unique identification number. If no questionnaire was received from the GP, the researcher asked the case manager to send a reminder. The case manager filled in a questionnaire with structured questions on the patient’s demographic data and care characteristics. For this paper we used age, sex and the main diagnosis. Furthermore, GPs completed a questionnaire containing structured questions regarding the GP’s characteristics and the care given to the patient, such as the number of contacts and place of death. If place of death was not available from the GP questionnaire, either it was obtained from the questionnaire the case manager filled in after the patient’s death, or the case manager was asked about the place of death in an open question by mail or phone; and the information was then recorded in the data management system for tracking questionnaires.

### Data analysis

In the study on standard GP care, the questions on place of death and preferred place of death were coded as ‘don’t know’ if they had not been filled in by the GP. In the study on case managers, information from the case manager on place of death was coded according to the categories used in the GP questionnaire.

To compare patients who received additional support from a case manager with patients who received the standard care from their GPs, logistic regression analysis was performed on all variables with the source of the data as dependent variable (Standard GP care = 0; Study on case managers = 1). Age was included as a covariate for adjusted odds ratios.

## Results

### Response

**Fig 1:**
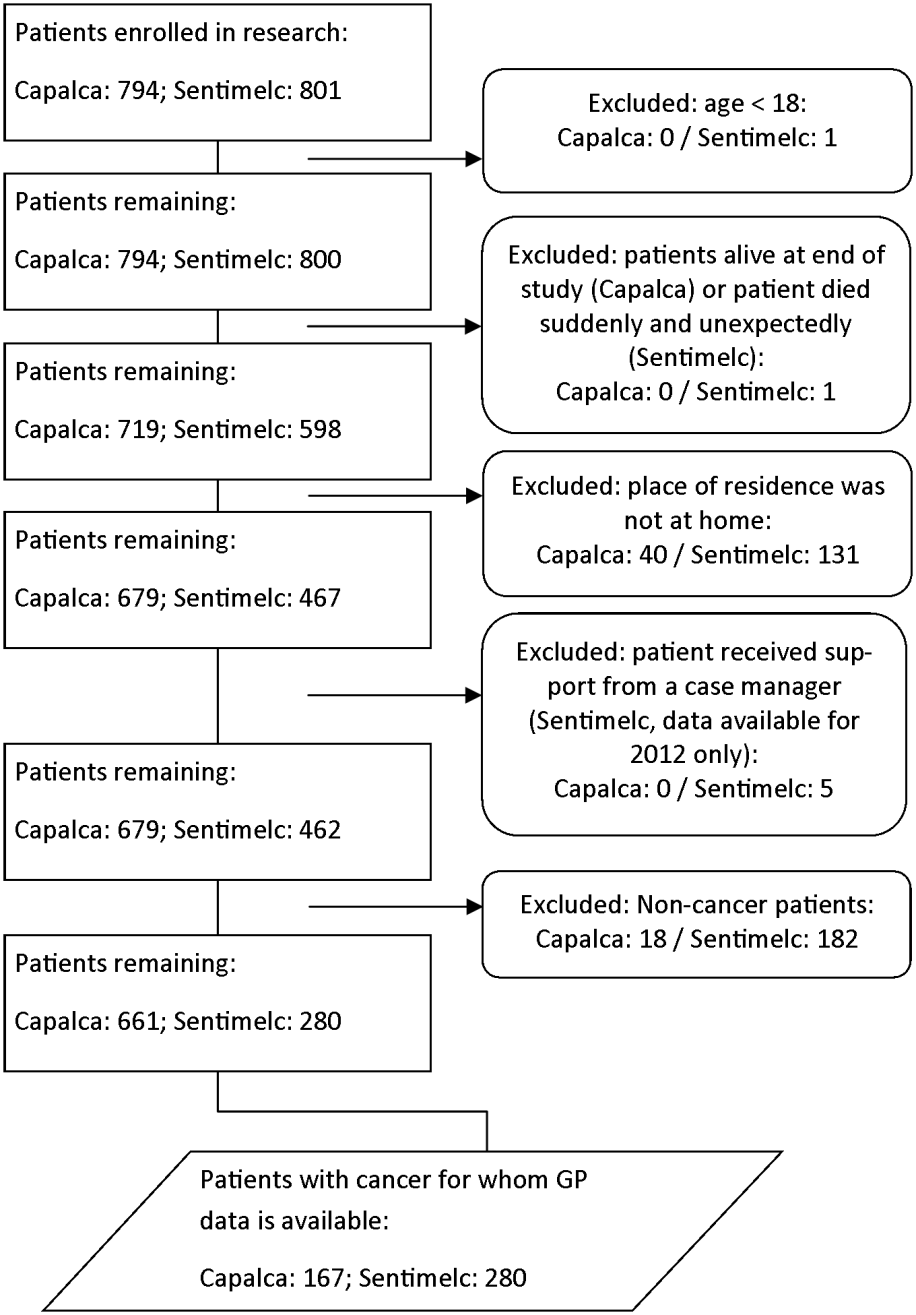
Flow chart of selection^†^. ^†^ Capalca = the study on case managers; Sentimelc = the study on standard GP care; GP = general practitioner.

A total of 794 adult patients were included in the study on case managers and 800 in the study on standard GP care. A flow chart of the effect of the exclusion criteria on the response is shown in Fig 1. For the comparison of care and outcome characteristics, data was available on 167 patients receiving support from a case manager and on 280 patients receiving standard GP care.

### Characteristics of cancer patients with and without additional support from a case manager

With regard to patients’ general characteristics (Table 1), patients referred to a case manager for additional support were younger (OR = 0.97; CI 0.95–0.98) compared with patients receiving the standard GP care.

**Table 1:** General characteristics of cancer patients with additional support from a case manager and patients receiving standard GP care.

	GP plus CM (n = 167)^†^	Standard GP care (n = 280)^†^	OR (CI) ^††^	OR (CI) Adjusted for age^††^	p value^§^
Age, mean (SD)	66 (12)	72 (12)	**0.97 (0.95–0.98)**		**<0.000**
Sex = female	78 (47)	124 (45)	1.09 (0.74–1.60)	0.98 (0.66–1.46)	0.927
Type of cancer:					
- lung	41 (25)	64 (26)	0.97 (0.62–1.53)	0.93 (0.58–1.48)	0.757
- colon	21 (13)	40 (16)	0.77 (0.44–1.37)	0.85 (0.47–1.52)	0.575
- breast	14 (9)	18 (7)	1.21 (0.58–2.50)	0.99 (0.47–2.12)	0.987
- hematologic or lymphatic	4 (3)	18 (7)	**0.32 (0.11–0.97)**	0.38 (0.12–1.15)	0.085
- prostate	10 (6)	15 (6)	1.02 (0.45–2.33)	1.42 (0.61–3.33)	0.422
- other	73 (45)	94 (38)	1.34 (0.90–2.00)	1.26 (0.84–1.90)	0.266

^†^ Values are numbers (percentages) unless stated otherwise. GP = general practitioner; CM = case manager. Total number of patients is 447. Missing values per variable: Age: no missing values, Sex: 2 missing values (Study on case managers 0; Standard GP care 2), Type of cancer: 35 missing values (Study on case managers 4; Standard GP care 31).

^††^ Dependent variables coded ‘Standard GP care’ = 0; ‘Study on case managers ‘ = 1. OR = Odds ratio; CI = 95% confidence interval. Confidence intervals not including the value 1 are considered statistically significant and are boldfaced.

^§^ Logistic regression analysis, adjusted for age

### Care characteristics of cancer patients with and without additional support from a case manager

Looking at the care and support provided to cancer patients (Table 2), after adjusting for age differences, patients referred to a case manager for additional support were more likely to have at least one contact with their GP in the last week of their lives (90% versus 84%; OR = 1.91; CI 1.04–3.52), had fewer contacts with their GP in the second and third months before death (1.4 versus 1.8 contacts; OR = 0.86; CI 0.74–0.99), and were more likely to have a palliative care consultant or consultation team involved in their care (24% versus 9%; OR = 3.23; CI 1.81–5.74) compared with patients receiving standard GP care.

**Table 2:** Characteristics of care and support for cancer patients with additional support from a case manager and patients receiving standard GP care.

	GP plus CM (n = 167) ^†^	standard GP care (n = 280) ^†^	OR (CI) ^§^	OR (CI) Adjusted for age^§^	p value^‡^
Number of patients with contact with the GP					
- contact in the last week	150 (90)	234 (84)	1.74 (0.96–3.14)	**1.91 (1.04–3.52)**	**0.038**
- contact in weeks 2–4	152 (91)	242 (86)	1.59 (0.85–2.99)	1.67 (0.87–3.20)	0.121
- contact in months 2–3	143 (86)	233 (83)	1.20 (0.71–2.05)	1.25 (0.73–2.16)	0.417
Number of contacts between GP and patient, mean (SD)^††^					
- in the last week	4.0 (2.9)	4.2 (2.7)	0.98 (0.91–1.06)	0.98 (0.90–1.05)	0.514
- in weeks 2–4	1.7 (0.8)	1.8 (1.2)	0.87 (0.71–1.07)	0.86 (0.70–1.07)	0.174
- in months 2–3	2.1 (1.4)	2.4 (1.8)	0.90 (0.79–1.03)	**0.86 (0.74–0.99)**	**0.032**
Involvement of home-care nurse, yes^§§^	120 (75)	106 (71)	1.22 (0.74–2.01)	1.41 (0.84–2.39)	0.197
Involvement of a palliative care consultant / consultation team in last 90 days, yes	37 (24)	23 (9)	**3.27 (1.86–5.76)**	**3.23 (1.81–5.74)**	**<0.000**

^†^ Values are numbers (percentages) unless stated otherwise. GP = general practitioner; CM = case manager. Total number of patients is 447. Missing values per variable between 0 and 32.

^††^ There are 63 patients (Standard GP care 46; Study on case managers 17) with no contacts in the final week, 53 patients (Standard GP care 38; Study on case managers 15) with no contacts in weeks 2 to 4, and 71 patients (Standard GP care 47; Study on case managers 24) with no contacts in months 2 and 3 who are excluded in this variable.

^§^ Dependent variables coded ‘Standard GP care’ = 0; ‘Study on case managers’ = 1. OR = Odds ratio; CI = 95% confidence interval. Confidence intervals not including the value 1 are considered statistically significant and are boldfaced.

^§§^ These data are not available for standard GP care from 2011. Study on case managers n = 167 and standard GP care 2012 n = 152.

^‡^ Logistic regression analysis, adjusted for age

### Care outcomes of cancer patients with and without additional support from a case manager

Looking at the outcomes of care for cancer patients (Table 3) after adjusting for age differences, the GP was more likely to know the preferred place of death (94% versus 72%; OR = 7.06; CI 3.47–14.36), the patient was more likely to have died at home (82% versus 69%; OR = 2.16; CI 1.33–3.51) and less likely to have died in hospital (7% versus 20%; OR = 0.26; CI 0.13–0.52), and more likely to have had no hospitalisations in the last 30 days of life (79% versus 69%; OR = 1.99; CI 1.12–3.56) and less likely to have had one hospitalisation (20% versus 30%; OR = 0.54; CI 0.30–0.96), if the patient had been referred to a case manager for additional support compared with patients receiving standard GP care.

**Table 3:** Preferred place of death, place of death, and number of transfers and hospitalisations of cancer patients with additional support from a case manager and patients receiving standard GP care.

	GP plus CM (n = 167) ^†^	Standard GP care (n = 280) ^†^	OR (CI) ^††^	OR (CI) Adjusted for age^††^	p value^§^
Preferred place of death is known by GP	157 (94)	202 (72)	**6.06 (3.04–12.09)**	**7.06 (3.47–14.36)**	**<0.000**
Number of patients who died at the preferred place of death	138 (88)	181 (91)	0.76 (0.39–1.50)	0.75 (0.37–1.52)	0.428
Place of death					
- at home or with carer	137 (82)	193 (69)	**2.01 (1.26–3.22)**	**2.16 (1.33–3.51)**	**0.002**
- hospice or palliative care unit	14 (8)	22 (8)	1.07 (0.53–2.14)	1.12 (0.55–2.29)	0.753
- hospital	12 (7)	55 (20)	**0.31 (0.16–0.61)**	**0.26 (0.13–0.52)**	**<0.000**
- care or nursing home	3 (2)	8 (3)	0.62 (0.16–2.36)	0.70 (0.17–2.87)	0.623
- other	1 (1)	0	NA	NA	
Number of transfers in last 30 days					
- none	62 (66)	171 (62)	1.18 (0.72–1.93)	1.33 (0.80–2.21)	0.270
- one	22 (23)	60 (22)	1.10 (0.63–1.91)	1.04 (0.59–1.84)	0.886
- two or more	10 (11)	44 (16)	0.63 (0.30–1.30)	0.53 (0.25–1.13)	0.099
Number of hospitalisations in last 30 days					
- none	74 (79)	189 (69)	1.68 (0.97–2.94)	**1.99 (1.12–3.56)**	**0.020**
- one	19 (20)	82 (30)	0.60 (0.34–1.05)	**0.54 (0.30–0.96)**	**0.037**
- two or more	1 (1)	4 (2)	0.73 (0.08–6.60)	0.33 (0.03–3.23)	0.343

^†^ Values are numbers (percentages) unless stated otherwise. GP = general practitioner; CM = case manager. Total number of patients is 447. Missing values per variable: Preferred place of death known: no missing values, Died at preferred place of death: 90 missing values (Study on case managers 10; Standard GP care 80), Place of death: 2 missing values (Study on case managers 0; Standard GP care 2), Number of transfers: 78 missing values (Study on case managers 73; Standard GP care 5), Number of hospitalisations: 78 missing values (Study on case managers 73; Standard GP care 5).

^††^ Dependent variables coded ‘Standard GP care’ = 0; ‘Study on case managers’ = 1. OR = Odds ratio; CI = 95% confidence interval. Confidence intervals not including the value 1 are considered statistically significant and are boldfaced.

^§^ Logistic regression analysis, adjusted for age

## Discussion

The GP was more likely to know the preferred place of death, and the place of death was more likely to be the home and less likely to be the hospital, for cancer patients referred to a case manager for additional support. Also, fewer hospitalisations occurred in the last 30 days of life, if a case manager was involved compared with patients receiving standard GP care. Cancer patients referred to a case manager for additional support were younger than patients receiving standard GP care. Also, they were more likely to have at least one contact with their GP in the last week of their lives, had fewer contacts with their GP in the months two and three before death, and were more likely to have a palliative care consultant or consultation team involved in their care compared with patients receiving standard GP care.

### More home deaths and fewer hospitalisations

The finding that a greater proportion of the patients receiving additional support from the case manager died at home and that they experienced fewer hospitalisations in the last 30 days of life is likely to be linked to the higher percentage (94%) of patients for whom the preferred place of death was known. For patients receiving palliative care from their GP, the percentage of patients with a known preferred place of death (72%) was similar to that of cancer patients in a previous study using Dutch Sentimelc data from 2005–2006 (70%) [24]. In that study, the preferred place was the same as the actual place of death for four-fifth of patients. In a comparison of four European countries (Belgium, Italy, Spain and the Netherlands), the percentage of patients whose GP knew their preferred place of death ranged from 27% (Italy) to 72% (the Netherlands); when known, the preference was met for 68% (Italy) to 92% (Spain) of patients [25].

Our findings are in line with a literature review in which specialised home palliative care increased the chance of dying at home [12]. Different models of specialised home care were included in the review study; our paper focusses on case managers who offer advice and support while the GP and home-care nurses continue to be main care providers. Underlying mechanisms should be further investigated, with attention to both the direct and indirect influences of the case manager on the care provided by the GP. The case manager can directly influence care provision by the GP, for instance by giving information to the GP about palliative care and supporting the GP in providing palliative care. The case manager can also indirectly influence care provision by the GP by encouraging and helping the patient to discuss palliative care options with their GP. Just getting a notification that a palliative care case manager is involved with the patient might trigger the GP’s awareness of the patient’s palliative care needs. End-of-life conversations between the GP and the patient occur more frequently when there is a palliative care treatment goal, and discussion of end-of-life issues is also associated with the GP being informed about the preferred place of death [26]. Hospital costs make up 40% of the total healthcare costs in the last six months of life [27]. An economic evaluation of the case management initiatives should be conducted to investigate whether the cost reduction due to fewer hospitalisations outweighs the cost of implementing case managers in palliative care.

### More patients with contacts in the last week of life, fewer contacts with the patient in months two and three

The number of contacts between the GP and the patients is lower in months two and three before the patients’ death when a case manager is involved, but the proportion of patients with contacts with their GP in the last week of life is higher. Although not significant, the proportion of patients with contacts with their GP is higher and the number of contacts between the GP and the patient is lower for all time frames when a case manager is involved. This could be an effect of coordinated care between the case manager and GP; it may be that the case manager and GP take turns in visiting the patient and therefore the GP will visit a patient less often when a case manager is involved. At the same time, the number of patients with some contact with the GP may be higher because the GP may be more aware that a patient has palliative care needs when a case manager is involved and the case manager may encourage the GP to visit a patient.

### Strengths and limitations of this study

This paper provides valuable information on care provision with and without the involvement of an additional case manager in primary palliative care. Information on standard GP care came from GPs who are part of the Sentinel network, which is designed to be nationally representative. The GPs in the study on case managers received a questionnaire from the case manager without any advance notice. The response rate for the study on case managers is low and the response may be skewed towards GPs with more positive experience of case managers and/or palliative care. Patients were not randomly assigned, and the patients with a case manager were younger than the patients receiving standard GP care. This limitation was allowed for by adjusting for age in the analyses. Other differences between the two groups, for example in the complexity of the disease may have been missed. Furthermore, the results may only be representative for mixed public–private healthcare systems with a strong primary care gatekeeper, which is the situation in the Netherlands. The case managers had an advisory role with respect to patients and other healthcare professionals. In other healthcare systems, task demarcation between generalist and specialist palliative care providers may be different, for instance because there are ‘hospice-at-home’ teams providing more comprehensive care that extends to prescribing medication and providing hand-on care. Also, care provision and outcomes may be different for patients with diagnoses other than cancer. Finally, further research is needed to better understand the experiences of patients, relatives, home-care nurses and GPs with the support provided by the case manager. A more detailed paper on the content of the support provided by the case manager will be published soon [18].

## Conclusion

Involvement of a case manager has added value in primary care in the model where generalist healthcare professionals cooperate with specialist palliative care providers. The percentage of patients who die at home is higher and the number of hospitalisations in the last 30 days of a patients’ life is lower when a case manager is involved offering advice and support.

## Supporting Information

S1 Dataset Dataset for PLOS ONE (IBM Statistics SPSS 20). (ZIP)
